# Supplementary material for: An ALE meta-analytic review of musical expertise
Source: Sci Rep. 2022 Jul 11;12:11726. doi: 10.1038/s41598-022-14959-4 (PMC9276732; doi:10.1038/s41598-022-14959-4)
Supplement: Supplementary file 1 — Supplementary Information. [file 41598_2022_14959_MOESM1_ESM.pdf]

SUPPLEMENTARY INFORMATION

An ALE meta-analytic review of musical expertise

Criscuolo, Antonio[1,2+], Pando-Naude, Victor[2+*], Bonetti, Leonardo[2, 3], Vuust, Peter[2] & Brattico, Elvira[2]

[1]Department of Neuropsychology & Psychopharmacology, Faculty of Psychology and Neuroscience, Maastricht University
[2]Center for Music in the Brain, Department of Clinical Medicine, Aarhus University & The Royal Academy of Music Aarhus/Aalborg, Denmark
[3]Center for Eudaimonia and Human Flourishing, Department of Psychiatry, University of Oxford, United Kingdom

+Shared first-authorship

*Corresponding author

Table of Contents

Search strings. 2

Supplementary Figure 1. PRISMA flowchart for literature search process. 3

PRISMA Checklist 4

Supplementary Table 1. Characteristics of MRI acquisition 6

Supplementary Table 2. Characteristics of MRI analyses. 8

Supplementary Table 3. Summary of MRI quality. 10

Supplementary Table 4. Meta-analytic connectivity modelling of regions-of-interest resulted from structural and functional ALE meta-analyses. 12

Supplementary Table 5. Functional characterization of brain regions resulted from structural and functional ALE meta-analyses 16

Supplementary Table 6. FSN robustness assessment of brain regions resulted from structural and functional ALE meta-analyses 19

Citations of included studies 20

Abbreviations 20

References 21

Search strings.

Last search: March, 2021

PubMed

("music"[MeSH Terms] OR "musician"[All Fields] OR "music"[All Fields]) AND ("education"[Subheading] OR "education"[All Fields] OR "training"[All Fields] OR "education"[MeSH Terms] OR ("motor skills"[MeSH Terms] OR ("motor"[All Fields] AND "skills"[All Fields]) OR "motor skills"[All Fields])) AND ("magnetic resonance imaging"[MeSH Terms] OR ("magnetic"[All Fields] AND "resonance"[All Fields] AND "imaging"[All Fields]) OR "magnetic resonance imaging"[All Fields] OR "plasticity"[All Fields] OR "functional connectivity"[All Fields] OR "resting-state"[All Fields] OR "structural"[All Fields] OR ("brain"[MeSH Terms] AND "activity"[All Fields]))

719 results

PsycInfo

(((Music OR musician) AND (education OR training)) AND magnetic resonance imaging)

155 results

Scopus

(TITLE-ABS-KEY (music) OR TITLE-ABS-KEY (musician)) AND (TITLE-ABS-KEY (education) OR TITLE-ABS-KEY (training)) AND TITLE-ABS-KEY (magnetic resonance imaging)

295 results

Supplementary Figure 1. PRISMA flowchart for literature search process.

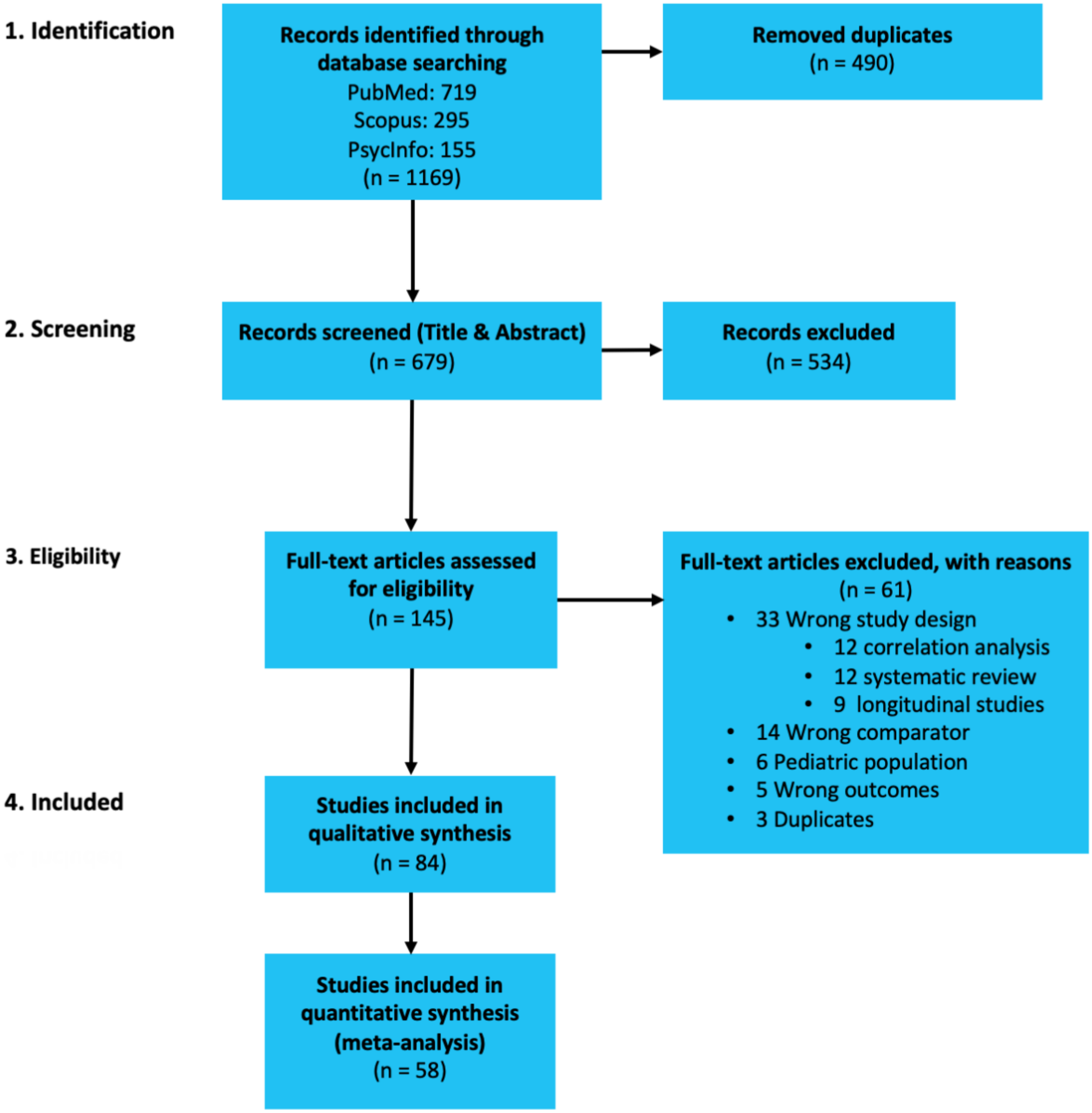

Supplementary Figure 1. PRISMA flowchart for literature search process.

PRISMA Checklist

SI = Supplementary Information

Section/topic	#	Checklist item	Reported on page #
**TITLE**			
Title	1	Identify the report as a systematic review, meta-analysis, or both.	1
**ABSTRACT**			
Structured summary	2	Provide a structured summary including, as applicable: background; objectives; data sources; study eligibility criteria, participants, and interventions; study appraisal and synthesis methods; results; limitations; conclusions and implications of key findings; systematic review registration number.	2
**INTRODUCTION**			
Rationale	3	Describe the rationale for the review in the context of what is already known.	3
Objectives	4	Provide an explicit statement of questions being addressed with reference to participants, interventions, comparisons, outcomes, and study design (PICOS).	3
**METHODS**			
Protocol and registration	5	Indicate if a review protocol exists, if and where it can be accessed (e.g., Web address), and, if available, provide registration information including registration number.	12
Eligibility criteria	6	Specify study characteristics (e.g., PICOS, length of follow-up) and report characteristics (e.g., years considered, language, publication status) used as criteria for eligibility, giving rationale.	12
Information sources	7	Describe all information sources (e.g., databases with dates of coverage, contact with study authors to identify additional studies) in the search and date last searched.	12
Search	8	Present full electronic search strategy for at least one database, including any limits used, such that it could be repeated.	SI
Study selection	9	State the process for selecting studies (i.e., screening, eligibility, included in systematic review, and, if applicable, included in the meta-analysis).	12
Data collection process	10	Describe method of data extraction from reports (e.g., piloted forms, independently, in duplicate) and any processes for obtaining and confirming data from investigators.	12
Data items	11	List and define all variables for which data were sought (e.g., PICOS, funding sources) and any assumptions and simplifications made.	12
Risk of bias in individual studies	12	Describe methods used for assessing risk of bias of individual studies (including specification of whether this was done at the study or outcome level), and how this information is to be used in any data synthesis.	12
Summary measures	13	State the principal summary measures (e.g., risk ratio, difference in means).	12
Synthesis of results	14	Describe the methods of handling data and combining results of studies, if done, including measures of consistency (e.g., I^2) for each meta-analysis.	12

Section/topic	#	Checklist item	Reported on page #
Risk of bias across studies	15	Specify any assessment of risk of bias that may affect the cumulative evidence (e.g., publication bias, selective reporting within studies).	12
Additional analyses	16	Describe methods of additional analyses (e.g., sensitivity or subgroup analyses, meta-regression), if done, indicating which were pre-specified.	12
**RESULTS**			
Study selection	17	Give numbers of studies screened, assessed for eligibility, and included in the review, with reasons for exclusions at each stage, ideally with a flow diagram.	4
Study characteristics	18	For each study, present characteristics for which data were extracted (e.g., study size, PICOS, follow-up period) and provide the citations.	4
Risk of bias within studies	19	Present data on risk of bias of each study and, if available, any outcome level assessment (see item 12).	4
Results of individual studies	20	For all outcomes considered (benefits or harms), present, for each study: (a) simple summary data for each intervention group (b) effect estimates and confidence intervals, ideally with a forest plot.	4
Synthesis of results	21	Present results of each meta-analysis done, including confidence intervals and measures of consistency.	4-6
Risk of bias across studies	22	Present results of any assessment of risk of bias across studies (see Item 15).	4
Additional analysis	23	Give results of additional analyses, if done (e.g., sensitivity or subgroup analyses, meta-regression [see Item 16]).	5
**DISCUSSION**			
Summary of evidence	24	Summarize the main findings including the strength of evidence for each main outcome; consider their relevance to key groups (e.g., healthcare providers, users, and policy makers).	7
Limitations	25	Discuss limitations at study and outcome level (e.g., risk of bias), and at review-level (e.g., incomplete retrieval of identified research, reporting bias).	9
Conclusions	26	Provide a general interpretation of the results in the context of other evidence, and implications for future research.	11
**FUNDING**			
Funding	27	Describe sources of funding for the systematic review and other support (e.g., supply of data); role of funders for the systematic review.	19

Supplementary Table 1. Characteristics of MRI acquisition

			Teslas	MRI-system	MRI-model	Head-coil	T1 sequence	TR (ms)	TE (ms)	Voxel size (mm)	T2*sequence	TR (ms)	TE (ms)	Voxel size (mm)	DTI sequence	TR (ms)	TE (ms)	Voxel size (mm)
1	Abdul-K	2011a	1.5	GE	Signa	quadrature	SPGR	34	9	-	-	-	-	-	-	-	-	-
2	Abdul-K	2011b	1.5	Siemens	Symphony	8-channel	MPRAGE	1660	3.04	1x1x1	-	-	-	-	DWI	10100	106	2x2x2
3	Acer	2018	1.5	Siemens	Aera	-	MPRAGE	1900	2.84	1x1x1	-	-	-	-	EPI	3500	83	1.8x1.8x3.5
4	Amunts	1997	1.5	Siemens	-	-	T1w	-	-	1x1x1.17	-	-	-	-	-	-	-	-
5	Angulo-P	2014	3	GE	Discovery	-	T1w	2300	3	1x1x1	EPI	3000	40	2x2x3	-	-	-	-
6	Bailey	2014	3	Siemens	Trio	32-channel	T1w	2300	2.98	1x1x1	-	-	-	-	-	-	-	-
7	Bangert	2006	1.5	GE	Signa	quadrature	SPGR	-	-	-	EPI	4500	40	-	-	-	-	-
8	Baumann	2007	3	Philips	Intera	8-channel	SPGR	20	2.3	0.98x0.98x0.75	EPI	2000	35	2.75x2.75x2.75	-	-	-	-
9	Bengtsson	2005	1.5	GE	Signa	-	T1w	24	6	0.86x0.86x2	-	-	-	-	EPI	18000	107	1.8x1.8x3
10	Berkowitz	2010	3	Philips	Intera	8-channel	MPRAGE	-	-	1x0.94x0.94	EPI	2500	35	-	-	-	-	-
11	Bermudez	2005	-	-	-	-	T1w	-	-	-	-	-	-	-	-	-	-	-
12	Bermudez	2009	1.5	Siemens	Vision	-	T1w	22	9.2	1x1x1	-	-	-	-	-	-	-	-
13	Bianchi	2017	3	Philips	Achieva	32-channel	T1w	6056	2.78	0.85	EPI	10000	30	3x3x3	-	-	-	-
14	Chen	2008	1.5	Siemens	Sonata	-	T1w	-	-	1x1x1	EPI	10000	50	5x5x5	-	-	-	-
15	Choi	2015	-	-	-	-	T1w	1900	2.52	1x1x1	-	-	-	-	-	-	-	-
16	De Manzano	2018	3	GE	Discovery	8-channel	IRPFSGE	6.7	2.9	1x1x1	-	-	-	-	-	-	-	-
17	Du	2017	3	Siemens	Magnetom	32-channel	MPRAGE	2300	2.98	1x1x1	EPI	636	30	3x3x3	-	-	-	-
18	Elmer	2012	3	Philips	Intera	8-channel	-	-	-	-	EPI	3000	35	1.72x1.72x4	-	-	-	-
19	Elmer	2013	3	Philips	Achieva	8-channel	T1w	8.06	3.7	0.94x0.94x0.94	-	-	-	-	-	-	-	-
20	Elmer	2016	3	Philips	Achieva	8-channel	-	-	-	-	-	-	-	-	EPI	13007	55	2x2x2
21	Gaab	2003	1.5	Siemens	Vision	-	T1w	-	-	1x1x1	custom	17000	-	-	-	-	-	-
22	Gaab	2006	3	GE	Signa	-	-	-	-	-	EPI	-	-	-	-	-	-	-
23	Gagnepain	2017	3	Philips	Achieva	-	T1w	20	4.6	1x1x1	EPI	500	80	2x1x1	-	-	-	-
25	Gaser	2003	1.5	Siemens	Vision	-	MPRAGE	-	-	1x1x1	-	-	-	-	-	-	-	-
24	Gärtner	2013	3	Siemens	Magnetom	12-channel	MPRAGE	2250	3.03	1x1x1	-	-	-	-	-	-	-	-
26	Giacosa	2016	3	Siemens	Trio	32-channel	-	9340	88	2x2x2	-	-	-	-	-	-	-	-
27	Groussard	2010	3	Philips	Achieva	-	FFE	20	4.6	1x1x1	EPI	2382	30	2.8x2.8x2.8	-	-	-	-
28	Groussard	2014	3	Philips	Achieva	-	FFE	20	4.6	1x1x1	-	-	-	-	-	-	-	-
29	Halwani	2011	3	GE	-	-	T1w	-	-	0.93x0.93x0.93	-	-	-	-	EPI	10000	86.9	2.5x2.5x2.6
30	Han	2009	3	GE	-	8-channel	SPGR	8.5	3.4	1x1x1	-	-	-	-	DWI	10000	70.8	-
31	Harris	2015	3	Philips	Intera	8-channel	T1w	-	-	-	EPI	16000	30	3.5x3.5x3.5	-	-	-	-
32	Haslinger	2004	1.5	Philips	Intera	birdcage	T1w	-	-	-	EPI	3000	50	3.59x3.59.5	-	-	-	-
33	Haslinger	2005	1.5	Philips	Intera	birdcage	T1w	-	-	-	EPI	3000	50	3.59x3.59.6	-	-	-	-
34	Herdener	2010	1.5	Siemens	-	-	MPRAGE	-	-	1.2x1x1	EPI	1850	61	5x5x5	-	-	-	-
35	Herdener	2014	-	-	-	-	-	-	-	-	EPI	-	-	-	-	-	-	-
36	Hernández	2019	3	Philips	Achieva	-	MPRAGE	8.4	3.8	0.9x0.89x0.8	-	-	-	-	-	-	-	-
37	Huang	2010	1.5	Siemens	Sonata	custom	FLASH	30	1.17	-	EPI	2000	60	-	-	-	-	-
38	Hutchinson	2003	1.5	Siemens	Vision	-	T1w	-	-	1x1x1	-	-	-	-	-	-	-	-
39	Imfeld	2009	3	GE	Signa	8-channel	-	-	-	-	-	-	-	-	EPI	8000	91	-
40	James	2014	3	Siemens	Trio	-	MPRAGE	1900	2.27	1x1x1	-	-	-	-	-	-	-	-
41	Karpati	2017	3	Siemens	Trio	32-channel	T1w	2300	2.98	1x1x1	-	-	-	-	-	-	-	-
42	Kleber	2010	1.5	Siemens	Vision	-	MPRAGE	-	-	-	EPI	10000	40	3	-	-	-	-
43	Kleber	2016	1.5	Siemens	Sonata	8-channel	MPRAGE	1300	3.19	-	-	-	-	-	-	-	-	-
44	Koelsch	2005	3	GE	-	-	T1w	-	-	1x1x1.5	-	6000	-	-	-	-	-	-
45	Koeneke	2004	1.5	GE	-	-	-	-	-	-	EPI	2000	40	3.125x3.126x6	-	-	-	-
46	Krings	2000	1.5	Philips	Gyroscan	-	T1w	-	-	-	EPI	456	35	-	-	-	-	-
47	Krishnan	2018	1.5	Siemens	Avanto	32-channel	T1w	2730	3.57	1x1x1	EPI	9500	50	2mm	-	-	-	-
48	Lee	2011	3	Siemens	Trio	-	T1w	2300	9.38	1x1x1	EPI	3000	40	3x3x3.3	-	-	-	-

49	Limb	2006	3	GE	Signa	quadrature	T1w	-	-	-	EPI	2000	30	6mm	-	-	-	-
50	Liu	2018	3	Siemens	Magnetom	-	T1w	1900	2.52	1x1x1	EPI	2000	30	3x3.4x3.4	-	-	-	-
51	Matsui	2013	1.5	GE	Signa	-	T1w	-	-	1x1x1.5	EPI	3000	55	4mm	-	-	-	-
52	Mathews	2020	3	Siemens	Trio	32-channel	T1w	2420	3.7	1x1x1	EPI	2000	27.92	2.35x2.35x2.5	-	-	-	-
53	Meister	2005	1.5	Philips	Gyroscan	quadrature	-	-	-	-	EPI	3587	50	5mm	-	-	-	-
54	Morrison	2003	1.5	GE	-	-	T1w	-	-	-	EPI	2500	50	3.5x3.5x5	-	-	-	-
55	Oechslin	2010	3	GE	Signa	8-channel	-	-	-	-	-	-	-	-	DWI	8000	91	-
56	Oechslin	2013	3	Siemens	Trio	-	MPRAGE	1900	2.27	1x1x1	EPI	18600	30	3.2x3.2x3.2	-	-	-	-
57	Oechslin	2018	3	Siemens	Trio	-	-	-	-	-	-	-	-	-	DWI	8300	83	2x2x2
58	Ohnishi	2001	1.5	Siemens	Magnetom	quadrature	-	-	-	-	EPI	3000	60	3.44x3.44x4	-	-	-	-
59	Ono	2015	3	Bruker	Medspec	birdcage	T1w	-	-	1x1x1	EPI	2000	30	3mm	-	-	-	-
60	Öztürk	2002	1.5	GE	Signa	-	T1w	500	20	-	-	-	-	-	-	-	-	-
61	Park	2014	3	Siemens	Magnetom	TIM	MPRAGE	2400	3.06	1x1x1	EPI	3000	30	3x3x4	-	-	-	-
62	Pau	2013	3	Siemens	Magnetom	12-channel	MPRAGE	-	-	1x1x1	EPI	2000	30	3x3x3	-	-	-	-
63	Petrini	2011	3	GE	Horizon	-	SPGR	-	-	1.5x0.9x0.9	EPI	2000	35	-	-	-	-	-
64	Rüber	2015	3	GE	-	-	T1w	-	-	0.93x0.93x1.5	-	-	-	-	-	-	-	-
65	Sakreida	2018	3	Siemens	Magnetom	8-channel	T1w	2040	5.57	1x1x1	EPI	2000	30	3x3x4.2	-	-	-	-
66	Sato	2015	3	Philips	Achieva	-	MPRAGE	15	3.3	1x1x1	-	-	-	-	-	-	-	-
67	Schlaffke	2020	3	Philips	Achieva	32-channel	MPRAGE	8.3	3.8	1x1x1	EPI	2500	35	2x2x3	-	-	-	-
68	Schlaug	1995a	1.5	-	-	-	-	-	-	-	-	-	-	-	-	-	-	-
69	Schlaug	1995b	1.5	-	-	-	-	-	-	-	-	-	-	-	-	-	-	-
70	Schlaug	2005	-	-	-	-	-	-	-	-	-	-	-	-	-	-	-	-
71	Schmithorst	2002	3	Bruker	Medspec	-	T1w	-	-	-	-	-	-	-	EPI	6000	87	-
72	Schmithorst	2003	3	Bruker	Biospec	-	T1w	-	-	-	EPI	3000	38	5mm	-	-	-	-
73	Schmithorst	2004	3	Bruker	Medspec	-	T1w	-	-	-	EPI	3000	38	5mm	-	-	-	-
75	Schneider	2002	1.5	Philips	Edge	-	T1w	-	-	1x1x1	-	-	-	-	-	-	-	-
74	Seung	2005	1.5	GE	Signa	-	T1w	-	-	-	EPI	3000	60	3.75x3.75x5	-	-	-	-
76	Sluming	2002	1.5	GE	Signa	quadrature	SPGR	34	9	-	-	-	-	-	-	-	-	-
77	Sluming	2007	1.5	GE	-	-	-	-	-	-	EPI	3000	40	5mm	-	-	-	-
78	Steele	2013	3	Siemens	Trio	32-channel	MPRAGE	2300	2.98	1x1x1	-	-	-	-	DWI	9340	88	2x2x2
79	Vaquero	2016	3	Siemens	Magnetom	-	MPRAGE	16	4.9	1x1x1	-	-	-	-	-	-	-	-
80	Vaquero	2020	3	Siemens	Magnetom	-	T1w	-	-	-	-	-	-	-	EPI	1000	90	2x2x2
81	Wang	2019	3	Siemens	Magnetom	-	MPRAGE	2300	3.24	1x1x1	-	-	-	-	-	-	-	-
82	Zarate	2005	1.5	-	-	-	T1w	-	-	-	-	10000	2.125	-	-	-	-	-
83	Zarate	2008	1.5	Siemens	Sonata	-	T1w	-	-	1x1x1	EPI	10000	85	5x5x5	-	-	-	-
84	Zuk	2014	3	Siemens	Trio	-	-	-	-	-	EPI	2000	30	3x3x4	-	-	-	-

GM, grey matter; WM, white matter; MRI, magnetic resonance imaging; FFE, fast field echo sequence; FLASH, fast low angle shot sequence; FSL, functional MRI of the brain software library; GE, gradient echo pulse; IR-FSPGR, fast spoiled gradient sequence with inversion preparation; MPRAGE, magnetization-prepared rapid acquisition with gradient echo sequence; MDEFT, modified driven equilibrium Fourier transform; SPGR, spoiled gradient recalled sequence; SPM, statistical parametric mapping; TFE, turbo field echo sequence; VBM, voxel-based morphometry[1,2].

Supplementary Table 2. Characteristics of MRI analyses.

			VBM Analysis Software	VBM Analysis Method	DTI Analysis Software	DTI Analysis Method	CT Analysis Software	CT Analysis Method	fMRI Analysis Method	fMRI Analysis software	Task	Stimuli	Control
1	Abdul-K	2011a	SPSS 16	VBM-ROI	-	-	-	-	-	-	-	-	-
2	Abdul-K	2011b	-	-	SPSS	FA	-	-	-	-	-	-	-
3	Acer	2018	VBM8	VBM-whole brain	DTIStudio	FA+MD	-	-	-	-	-	-	-
4	Amunts	1997	-	VBM-ROI	-	-	-	-	-	-	-	-	-
5	Angulo-P	2014	-	-	-	-	-	-	GLM	FSL	listen	music excerpts	speech
6	Bailey	2014	FSL	VBM-whole brain	-	-	FSL	CT	-	-	-	-	-
7	Bangert	2006	-	-	-	-	-	-	GLM	SPM99	listen+play	piano	rest
8	Baumann	2007	-	-	-	-	-	-	GLM	SPM2	listen+tapping	music excerpts	rest
9	Bengtsson	2005	SPM99	VBM-whole brain	SPM-99	FA	-	-	-	-	-	-	-
10	Berkowitz	2010	-	-	-	-	-	-	GLM	BrainVoyager	play music	piano	patterns
11	Bermudez	2005	-	VBM-whole brain	-	-	-	-	-	-	-	-	-
12	Bermudez	2009	CIVET	VBM-whole brain	-	-	CIVET	MACACC	-	-	-	-	-
13	Bianchi	2017	-	-	-	-	-	-	GLM	SPM8	listen+rate	tones	rest
14	Chen	2008	-	-	-	-	-	-	GLM	fMRISTAT	listen+tapping	rhythm	rest
15	Choi	2015	Brainvoyager	-	-	-	BrainVoyager	CT	-	-	-	-	-
16	De Manzano	2018	SPM12	VBM-ROI	Mrtrix3	FA	FreeSurfer	LME	-	-	-	-	-
17	Du	2017	-	-	-	-	-	-	GLM	AFNI	listen+rate	speech	noise
18	Elmer	2012	-	-	-	-	-	-	GLM	SPM8	listen+rate	speech	noise
19	Elmer	2013	-	-	-	-	FreeSurfer	-	-	-	-	-	-
20	Elmer	2016	-	-	FSL	FA	-	-	-	-	-	-	-
21	Gaab	2003	-	-	-	-	-	-	GLM	SPM99	listen+rate	tones	motor
22	Gaab	2006	-	-	-	-	-	-	GLM	SPM2	listen+play	tones	-
23	Gagnepain	2017	-	-	-	-	-	-	GLM	SPM8	listen+rate	melodies	proverbs
25	Gaser	2003	SPM99	VBM-whole brain	-	-	-	-	-	-	-	-	-
24	Gärtner	2013	SPM8	DBM-ROI	-	-	-	-	-	-	-	-	-
26	Giacosa	2016	-	-	FSL	TBSS	-	-	-	-	-	-	-
27	Groussard	2010	SPM5	VBM-whole brain	-	-	-	-	GLM	SPM5	-	melodies	unfamiliar melodies
28	Groussard	2014	SPM12	VBM-whole brain	-	-	-	-	-	-	-	-	-
29	Halwani	2011	-	-	FSL	FA	-	-	-	-	-	-	-
30	Han	2009	SPM2	VBM-whole brain	AFNI	FA	-	-	-	-	-	-	-
31	Harris	2015	-	-	-	-	-	-	GLM	SPM5	listen+imagine	piano	score-reading
32	Haslinger	2004	-	-	-	-	-	-	GLM	SPM99	play music	piano	rest
33	Haslinger	2005	-	-	-	-	-	-	GLM	SPM99	audiovisual	piano	rest
34	Herdener	2010	-	-	-	-	-	-	GLM	BrainVoyager	-	tones	mismatch
35	Herdener	2014	-	-	-	-	-	-	GLM	BrainVoyager	listen+imagine	tones	mismatch
36	Hernández	2019	SPM12	VBM-ROI	-	-	-	-	-	-	-	-	-
37	Huang	2010	-	-	-	-	-	-	GLM	AFNI	listen+rate	words	-
38	Hutchinson	2003	custom	VBM-ROI	-	-	-	-	-	-	-	-	-
39	Imfeld	2009	-	-	SPM5	FA	-	-	-	-	-	-	-
40	James	2014	SPM8	VBM-whole brain	-	-	-	-	-	-	-	-	-
41	Karpati	2017	CIVET	VBM-whole brain	-	-	-	-	-	-	-	-	-
42	Kleber	2010	-	-	-	-	-	-	GLM	SPM5	singing	-	rest
43	Kleber	2016	SPM5	VBM-whole brain	-	-	-	-	-	-	-	-	-
44	Koelsch	2005	-	-	-	-	-	-	GLM	SPM99	listen	tones	deviants
45	Koeneke	2004	-	-	-	-	-	-	GLM	SPM99	play	-	rest
46	Krings	2000	-	-	-	-	-	-	GLM	-	tapping	-	rest

47	Krishnan	2018	FSL	VBM-whole brain	-	-	-	-	GLM	SPM8	listen	beatbox/guitar	rest
48	Lee	2011	-	-	-	-	-	-	GLM	SPM8	audiovisual	sentences/piano	rest
49	Limb	2006	-	-	-	-	-	-	GLM	SPM99	listen	rhythm	rest
50	Liu	2018	-	-	-	-	-	-	GLM	SPM8	listen+rate	music excerpts	rest
51	Matsui	2013	-	-	-	-	-	-	GLM	SPM8	listen	music excerpts	scrambled
52	Mathews	2020	-	-	-	-	-	-	GLM	SPM12	listen+rate	piano	rest
53	Meister	2005	-	-	-	-	-	-	GLM	SPM99	play music	-	rest
54	Morrison	2003	-	-	-	-	-	-	GLM	MEDx	listen	music excerpts	speech
55	Oechslin	2010	-	-	SPM5	FA+MD	-	-	-	-	-	-	-
56	Oechslin	2013	-	-	-	-	-	-	GLM	SPM8	listen+rate	music excerpts	scrambled
57	Oechslin	2018	-	-	FSL	FA	-	-	-	-	-	-	-
58	Ohnishi	2001	-	-	-	-	-	-	GLM	SPM99	listen	music excerpts	rest
59	Ono	2015	-	-	-	-	-	-	GLM	SPM8	tapping	conductors	metronome
60	Öztürk	2002	-	-	-	-	-	-	-	-	-	-	-
61	Park	2014	-	-	-	-	-	-	GLM	SPM8	listen+rate	music excerpts	noise
62	Pau	2013	-	-	-	-	-	-	GLM	SPM5	tapping	tones	noise
63	Petrini	2011	-	-	-	-	-	-	GLM	BrainVoyager	listen+rate	drums	rest
64	Rüber	2015	-	-	FSL	FA	-	-	-	-	-	-	-
65	Sakreida	2018	-	-	-	-	-	-	GLM	SPM8	tapping	rhythm	rest
66	Sato	2015	SPM12	VBM-whole brain	-	-	-	-	-	-	-	-	-
67	Schlaffke	2020	-	-	ExploreDTI	FA+MD	-	-	GLM	SPM8	tapping	drums	rest
68	Schlaug	1995a	-	-	-	-	-	-	-	-	-	-	-
69	Schlaug	1995b	-	-	-	-	-	-	-	-	-	-	-
70	Schlaug	2005	-	-	-	-	-	-	-	-	-	-	-
71	Schmithorst	2002	-	-	IDL	FA	-	-	-	-	-	-	-
72	Schmithorst	2003	-	-	-	-	-	-	GLM	IDL	listen	melodies	rest
73	Schmithorst	2004	-	-	-	-	-	-	GLM	IDL	math	visual	rest
75	Schneider	2002	Brainvoyager	VBM-ROI	-	-	-	-	-	-	-	-	-
74	Seung	2005	-	-	-	-	-	-	GLM	SPM99	listen	music excerpts	rest
76	Sluming	2002	SPM99	VBM-whole brain	-	-	-	-	-	-	-	-	-
77	Sluming	2007	-	-	-	-	-	-	GLM	SPM99	visual	drawing	rest
78	Steele	2013	-	-	FSL	FA	-	-	-	-	-	-	-
79	Vaquero	2016	SPM8	VBM-whole brain	-	-	-	-	-	-	-	-	-
80	Vaquero	2020	-	-	FSL	FA+RD	-	-	-	-	-	-	-
81	Wang	2019	SPM12	VBM-whole brain	-	-	-	-	-	-	-	-	-
82	Zarate	2005	-	-	-	-	-	-	-	-	sing	-	-
83	Zarate	2008	-	-	-	-	-	-	GLM	fMRISTAT	sing	vocal waves	noise
84	Zuk	2014	-	-	-	-	-	-	-	-	listen+rate	sounds	-

fMRI, functional magnetic resonance imaging; VBM, voxel-based morphometry; DTI, diffusion tensor imaging; CT, cortical thickness; ROI, region-of-interest; FSL, functional MRI of the brain software library; MPRAGE, magnetization-prepared rapid acquisition with gradient echo sequence; SPGR, spoiled gradient recalled sequence; SPM, statistical parametric mapping; FA, fractional anisotropy.

Supplementary Table 3. **Summary of MRI quality.**

															Main outcomes	
			MRI design described	Age reported	Sample gender reported	Sample handedness reported	Matched control group	Ethics approval reported	Image acquisition described	Image processing described	Statistical MRI-analysis described	Software package specified	Multiple comparison correction described	Figures and tables	M > NM	NM > M
1	Abdul-K	2011a	Y	Y	Y	Y	a,b,c	Y	Y	Y	Y	Y	Y	Y	lIFG	-
2	Abdul-K	2011b	Y	Y	Y	Y	a,b,c	Y	Y	Y	Y	Y	Y	Y	rSCP, rMCP, rCRBL	-
3	Acer	2018	Y	Y	Y	Y	a,b	Y	Y	Y	Y	Y	N	Y	rCST, rCRBL, lCRBL, SMG, AnG, lSPL, lIPL, MidTG	-
4	Amunts	1997	Y	Y	Y	Y	a,b,c	N	Y	U	U	U	U	Y	PreCG	-
5	Angulo-P	2014	Y	Y	Y	Y	a,b,c	Y	Y	Y	Y	Y	Y	Y	PT	-
6	Bailey	2014	Y	Y	N	Y	c	Y	Y	Y	Y	Y	Y	Y	PMC, SI	-
7	Bangert	2006	Y	Y	Y	Y	a,b,c	N	Y	Y	Y	Y	Y	Y	MidTG, SFG, SRG, PreCG, IFG, IPL, HIPP, DLPFC, SMG, ACC,	-
8	Baumann	2007	Y	Y	Y	Y	b,c,e	Y	U	Y	Y	Y	N	Y	PMC, SMA	-
9	Bengtsson	2005	Y	Y	Y	Y	a,b,c	Y	Y	U	Y	Y	Y	Y	IC	-
10	Berkowitz	2010	Y	U	U	N	a	N	Y	Y	Y	Y	Y	Y	rTPJ	-
11	Bermudez	2005	N	N	N	N	-	N	U	N	U	N	Y	Y	STG, PT	-
12	Bermudez	2009	Y	Y	Y	Y	a,b,c	Y	Y	Y	Y	N	Y	Y	HG, PreCG, IFG	-
13	Bianchi	2017	Y	Y	Y	Y	a,b,c	Y	Y	Y	Y	Y	Y	Y	STG, HG, PP, IFG, PreCG, FusG, CRBL, IF, INS	-
14	Chen	2008	Y	Y	N	Y	c	Y	Y	Y	Y	Y	N	Y	DLPFC, IFG, CRBL	-
15	Choi	2015	Y	Y	Y	N	a,b	Y	U	Y	Y	Y	Y	Y	SI (lips)	SI (tongue)
16	De Manzano	2018	Y	Y	Y	N	a,b	Y	Y	Y	Y	Y	Y	Y	STG, CRBL, WM	-
17	Du	2017	Y	Y	Y	Y	a,b,c	Y	Y	Y	Y	Y	Y	Y	AnG, MidTG, IFG,	CRBL
18	Elmer	2012	Y	Y	Y	Y	a,b,c	Y	Y	Y	Y	Y	Y	Y	PT, PMC	-
19	Elmer	2013	Y	Y	Y	Y	a,b,c,d	Y	Y	Y	Y	Y	Y	Y	PT	-
20	Elmer	2016	Y	Y	Y	Y	a,b,c,d	Y	Y	Y	Y	Y	Y	Y	CC	-
21	Gaab	2003	Y	Y	Y	Y	a,b,c	N	Y	Y	Y	Y	Y	Y	PT, SMG, SPL, SPL, IFG	PT, CRBL, HIPP
22	Gaab	2006	Y	Y	Y	Y	a,b	N	U	N	Y	Y	Y	Y	-	lIFG, MidFG, ACC, IPL
23	Gagnepain	2017	Y	Y	Y	Y	a,b,c	Y	Y	Y	Y	Y	Y	Y	HIPP	-
25	Gaser	2003	Y	Y	Y	Y	a,b,c,d	Y	Y	U	U	Y	Y	Y	ITG, M1, SPL, HG, IFG, MidFG, CRBL	-
24	Gärtner	2013	Y	Y	Y	Y	a,b,c	Y	Y	Y	Y	Y	N	Y	CC, CST, RN, EnC, THA	-
26	Giacosa	2016	Y	Y	Y	N	a,b	Y	Y	Y	Y	Y	Y	Y	CST, IFOF, SFL, ILF	-
27	Groussard	2010	Y	Y	Y	Y	a,b,c,d	Y	Y	Y	Y	Y	N	Y	HIPP, CalC, LG, OFC, MCC, STG, CRBL	-
28	Groussard	2014	Y	Y	Y	Y	a,b,c	Y	Y	Y	Y	Y	N	Y	HIPP, SMA, SFG, MidFG, PCC, INS, STG	-
29	Halwani	2011	Y	Y	N	N	a	Y	Y	Y	Y	Y	Y	Y	AF	-
30	Han	2009	Y	Y	Y	Y	a,b,c	Y	Y	Y	Y	Y	N	Y	M1, SI, CRBL, IFG, IC, MB	OFC, ACC
31	Harris	2015	Y	Y	Y	Y	a,b,c	Y	Y	Y	Y	Y	Y	Y	PMC, SMG, PCC, STG, STS	-
32	Haslinger	2004	Y	Y	Y	Y	a,b,c	Y	Y	Y	Y	Y	Y	Y	-	PMC, ACC, CRBL ITG, LG, CAU, SMA MidFG, PMC
33	Haslinger	2005	Y	Y	Y	Y	a,b,c	Y	Y	Y	Y	Y	Y	Y	PMC, SMA, IFG, MidFG, STG, M1, S1	-
34	Herdener	2010	Y	Y	Y	N	a,b	Y	Y	Y	Y	Y	Y	Y	HIPP, PCN, INS	-
35	Herdener	2014	Y	Y	Y	N	a,b	Y	N	N	U	Y	Y	Y	SMG, INS	-
36	Hernández	2019	Y	Y	Y	N	a,b	Y	Y	Y	Y	Y	Y	Y	CAU	-
37	Huang	2010	Y	Y	Y	Y	a,b,c,d	Y	Y	Y	Y	Y	Y	Y	LG, IFG, HIPP, AMYG, MedFG.	-
38	Hutchinson	2003	Y	Y	Y	Y	a,b,c	Y	U	U	Y	Y	Y	Y	CRBL	-
39	Imfeld	2009	Y	Y	Y	Y	a,c	Y	Y	Y	Y	Y	N	Y	CST	-
40	James	2014	Y	Y	N	Y	a,c	Y	Y	Y	Y	Y	Y	Y	FusG, OFC, CRBL, IFG, IPL, HG	PostCG, PCN, CAU
41	Karpati	2017	Y	Y	Y	N	a,b	Y	Y	Y	Y	Y	Y	Y	STG, STS, MTG	-
42	Kleber	2010	Y	Y	Y	Y	c	Y	Y	Y	Y	Y	Y	Y	M1, SI, SMA, DLPFC, TP, GP, CRBL , PCN, PUT, THA	-
43	Kleber	2016	Y	Y	Y	Y	a,b,c	Y	Y	Y	Y	Y	Y	Y	SI, SMG, SII, STG, HIPP, CAU	-
44	Koelsch	2005	Y	Y	Y	Y	a,b,c	Y	Y	Y	Y	Y	N	Y	STG, SMG, PL, IFG	-
45	Koeneke	2004	Y	Y	Y	Y	a,b,c	Y	Y	Y	Y	Y	Y	Y	rCRBL	lCRBL, VER
46	Krings	2000	Y	Y	Y	Y	a,b,c	N	U	N	U	N	N	Y	-	S1, SMA, PMC, SPL

47	Krishnan	2018	Y	Y	Y	N	a,b	Y	Y	Y	Y	Y	Y	Y	CRBL, IFG, IPC	IFG, ITG, PostCG, SMA, CRBL
48	Lee	2011	Y	Y	N	N	a	N	Y	Y	Y	Y	Y	Y	STS, MTG, PMC, CRBL	-
49	Limb	2006	Y	Y	Y	Y	a,b,c	Y	Y	Y	Y	Y	N	Y	MTG, AnG, SMG, FO, MidFG, SFG	STG, SMG, PCN, Cuneus, PreCG, GP, PUT
50	Liu	2018	Y	N	Y	Y	a,c	Y	Y	N	Y	Y	Y	Y	IPL, STG, HG, PCN, MTG, PCC	-
51	Matsui	2013	Y	Y	Y	Y	a,b,c	Y	Y	Y	Y	Y	Y	Y	MTG, STG	STG
52	Mathews	2020	Y	Y	Y	Y	a	Y	Y	Y	Y	Y	Y	Y	PreCG, SMA, IFG, HG, CAU, STG, SMG	-
53	Meister	2005	Y	Y	Y	Y	b,c	Y	Y	Y	Y	Y	U	Y	FP	-
54	Morrison	2003	Y	Y	Y	Y	b	Y	Y	Y	Y	Y	Y	Y	STG	-
55	Oechslin	2010	Y	Y	Y	Y	a,b	Y	Y	Y	Y	Y	Y	Y	AF	-
56	Oechslin	2013	Y	Y	N	Y	a,c	N	Y	Y	Y	Y	Y	Y	ACC, SMG, SMA, PO, PCN, PostCG	-
57	Oechslin	2018	Y	Y	Y	Y	a,b,c,d	N	Y	Y	Y	Y	Y	Y	rVentral stream	-
58	Ohnishi	2001	Y	Y	Y	Y	a,b,c	Y	Y	Y	Y	Y	Y	Y	STG, MidFG	-
59	Ono	2015	Y	Y	Y	Y	a,b,c	Y	Y	Y	Y	Y	Y	Y	SFG	-
60	Öztürk	2002	Y	Y	Y	Y	b,c	Y	U	U	Y	N	N	Y	CC	-
61	Park	2014	Y	Y	Y	Y	a,b,c	Y	Y	Y	Y	Y	N	Y	MidFG, PreCG, SFG, SMG, PostCG, IPL	-
62	Pau	2013	Y	Y	Y	Y	a,b,c	Y	Y	Y	Y	Y	Y	Y	SI, M1, SMA, PMC, SPL, IFG, CRBL, DLPFC, OL, INS, STG, MTG	S1, M1, SMA, PMC, IPL, IFG, CRBL, PUT, DLPFC, INS, STG, MTG
63	Petrini	2011	Y	Y	Y	Y	a,b,c	N	Y	Y	Y	Y	Y	Y	-	MidFG
64	Rüber	2015	Y	Y	Y	Y	a,b,c	Y	Y	Y	Y	Y	Y	Y	M1	-
65	Sakreida	2018	Y	Y	Y	Y	a,b	Y	Y	Y	Y	Y	Y	Y	IPL, SMG, ITG,, MTG, MOG, IFG, MidFG, MidTG, SMA, SFG, PCN, SPL, PUT, INS, CRBL	IPL, AnG, SMG, CUN, IFG
66	Sato	2015	Y	Y	Y	Y	a,b,c	Y	Y	Y	Y	Y	N	Y	MOG, IFG, LG, STG, PCN, CAU, SPL, TP	CAU
67	Schlaffke	2020	Y	Y	Y	Y	a,b,c	Y	Y	Y	Y	Y	Y	Y	CC	-
68	Schlaug	1995a	Y	Y	Y	Y	a,b	N	N	N	Y	N	Y	Y	CC	-
69	Schlaug	1995b	Y	Y	Y	Y	a,b	N	N	N	Y	N	Y	Y	PT	-
70	Schlaug	2005	N	N	N	N	-	N	N	N	N	N	N	Y	SI, M1, PMC, SPL, HG, CRBL, IFG	-
71	Schmithorst	2002	Y	Y	N	N	a	Y	Y	Y	Y	Y	Y	Y	CC	-
72	Schmithorst	2003	Y	Y	Y	N	a	Y	Y	Y	Y	Y	Y	Y	FusG, LG	ACC, SFG
73	Schmithorst	2004	Y	Y	Y	N	a	Y	Y	Y	Y	Y	Y	Y	FusG, MedFG	IOG, MOG, ThA, OFG, IPL
75	Schneider	2002	Y	Y	Y	Y	a,b,c	Y	Y	Y	Y	Y	N	Y	HG	-
74	Seung	2005	Y	Y	Y	Y	a,b,c	Y	Y	Y	Y	Y	Y	Y	STG, MTG, IFG, SMG	PCN, LG
76	Sluming	2002	Y	Y	Y	Y	a,b,c	N	Y	Y	Y	Y	Y	Y	IFG	-
77	Sluming	2007	Y	Y	Y	Y	a,b,c	Y	Y	Y	Y	Y	Y	Y	IFG, AnG, ACC	PCN, AnG, SPL, PrecG, SMA
78	Steele	2013	Y	Y	Y	Y	a,c	Y	Y	Y	Y	Y	Y	Y	CC	-
79	Vaquero	2016	Y	Y	Y	Y	a,c	Y	Y	Y	Y	Y	Y	Y	PUT, AMYG, CS, LG, PUT, THA, STG	SMG, STG, PostCG
80	Vaquero	2020	Y	Y	Y	Y	a,b,c,d	Y	Y	Y	Y	Y	Y	Y	AF	-
81	Wang	2019	Y	Y	Y	Y	a,b,c,d	Y	Y	Y	Y	Y	Y	Y	-	INS
82	Zarate	2005	U	N	Y	N	a,b	N	Y	N	N	N	N	Y	ACC, STS, INS; PUT, SMA	ACC, IPL
83	Zarate	2008	Y	Y	Y	Y	a	Y	Y	Y	Y	Y	Y	Y	GP; CRBLV	ACC
84	Zuk	2014	Y	Y	Y	Y	a,b,c	Y	Y	Y	Y	Y	Y	Y	SMA, PFC	-

Y, yes; N, no; U, unclear.

* a, age; b, sex; c, handedness; d, education.

MRI guidelines[1,2]

Supplementary Table 4. Meta-analytic connectivity modelling of regions-of-interest resulted from structural and functional ALE meta-analyses.

Cluster Number	Volume (mm3)	MNI coordinates x	y	z	ALE	P	Z	Label (Side region BA)
**a. STRUCTURAL ALE ROIs**								
M>NM (GM)								
1. STG-R BA13: 992 foci, 46 experiments, 593 subjects (x=50, y=-20, z=8)								
1	24288	-58	-22	8	5E-02	2E-15	7.8	L Superior Temporal Gyrus BA41
		-50	-16	0	5E-02	7E-15	7.7	L Superior Temporal Gyrus BA22
		-42	-26	10	5E-02	7E-14	7.4	L Transverse Temporal Gyrus BA41
		-38	-32	14	5E-02	5E-13	7.1	L Superior Temporal Gyrus BA41
		-50	10	2	4E-02	6E-10	6.1	L Insula BA13
		-52	-38	18	4E-02	1E-09	6.0	L Insula BA13
		-32	20	4	4E-02	1E-09	6.0	L Insula BA13
		-58	-8	-2	3E-02	6E-07	4.8	L Superior Temporal Gyrus BA22
		-36	2	4	2E-02	2E-04	3.6	L Claustrum
2	14968	50	-20	8	2E-01	0E+00	20.0	R Superior Temporal Gyrus BA13
		62	-28	4	4E-02	4E-11	6.5	R Superior Temporal Gyrus BA22
		60	-4	-6	3E-02	6E-08	5.3	R Superior Temporal Gyrus BA22
		62	-4	16	2E-02	2E-04	3.5	R Precentral Gyrus BA4
3	5520	48	10	2	4E-02	7E-11	6.4	R Precentral Gyrus BA44
		38	22	0	3E-02	4E-08	5.4	R Insula BA13
		50	20	-6	2E-02	2E-05	4.1	R Inferior Frontal Gyrus BA47
4	3528	-4	0	62	4E-02	4E-09	5.8	L Medial Frontal Gyrus BA6
		0	8	52	3E-02	5E-06	4.4	L Medial Frontal Gyrus BA6
		2	16	40	2E-02	9E-05	3.8	L Cingulate Gyrus BA32
5	1856	-30	-56	-28	3E-02	3E-07	5.0	L Culmen
		-14	-62	-18	3E-02	7E-06	4.3	L Declive
		-24	-66	-18	2E-02	1E-04	3.6	L Declive
6	1536	-20	6	4	3E-02	7E-08	5.3	L Lentiform Nucleus
		-12	-2	12	2E-02	5E-05	3.9	L Caudate
7	1472	-12	-18	2	3E-02	3E-08	5.4	L Thalamus
2. STG-L BA41: 1428 foci, 71 experiments, 961 subjects (x=-56, y=-20, z=2)								
1	25008	-56	-20	2	3E-01	0E+00	25.6	L Superior Temporal Gyrus BA41
		-56	-2	-8	5E-02	6E-12	6.8	L Superior Temporal Gyrus BA22
		-40	-34	14	3E-02	3E-07	5.0	L Transverse Temporal Gyrus BA41
		-36	22	4	3E-02	2E-06	4.6	L Insula BA13
		-46	22	-10	3E-02	6E-06	4.4	L Inferior Frontal Gyrus BA47
		-34	26	-8	2E-02	7E-05	3.8	L Insula BA13
		-56	6	10	2E-02	7E-05	3.8	L Precentral Gyrus BA6
		-54	18	8	2E-02	2E-04	3.5	L Inferior Frontal Gyrus BA44
		-58	-4	20	2E-02	2E-04	3.5	L Postcentral Gyrus BA4
		-58	-8	26	2E-02	3E-04	3.4	L Precentral Gyrus BA4
		-60	-8	14	2E-02	4E-04	3.4	L Precentral Gyrus BA43
2	19728	60	-20	2	1E-01	5E-33	11.9	R Superior Temporal Gyrus BA41
		54	10	-14	4E-02	3E-10	6.2	R Superior Temporal Gyrus BA22
3	3416	36	22	-6	4E-02	3E-09	5.8	R Insula
		46	24	10	3E-02	5E-07	4.9	R Inferior Frontal Gyrus BA45
4	2568	-46	12	24	3E-02	7E-07	4.8	L Inferior Frontal Gyrus BA9
		-54	22	20	3E-02	5E-06	4.4	L Inferior Frontal Gyrus BA9
		-40	6	30	2E-02	7E-05	3.8	L Precentral Gyrus BA6
		-42	24	20	2E-02	4E-04	3.4	L Middle Frontal Gyrus BA46
5	2456	54	0	46	4E-02	1E-09	6.0	R Precentral Gyrus BA4
		54	6	36	3E-02	3E-06	4.5	R Precentral Gyrus BA6
6	1920	-50	-6	46	4E-02	3E-10	6.2	L Precentral Gyrus BA4
		-46	0	54	2E-02	2E-04	3.6	L Precentral Gyrus BA6
7	1856	0	4	62	3E-02	1E-07	5.1	L Medial Frontal Gyrus BA6
3. PostCG-R BA2: 437 foci, 22 experiments, 310 subjects (x=54, y=-22, z=44)								
1	5416	-2	4	50	3E-02	2E-09	5.9	L Medial Frontal Gyrus BA6
		-8	12	36	2E-02	1E-05	4.2	L Cingulate Gyrus BA24
2	5312	56	-22	44	1E-01	1E-45	14.2	R Postcentral Gyrus BA2
3	4744	-42	-28	54	3E-02	8E-10	6.0	L Inferior Parietal Lobule BA40
		-34	-18	64	3E-02	4E-09	5.8	L Precentral Gyrus BA4
4	1528	56	12	36	3E-02	1E-08	5.6	R Middle Frontal Gyrus BA9

		50	8	26	1E-02	4E-04	3.3	R Inferior Frontal Gyrus BA9
5	1432	10	-14	6	3E-02	2E-08	5.5	R Thalamus
6	1376	56	14	-6	3E-02	1E-07	5.2	R Superior Temporal Gyrus BA22
7	1376	64	-22	18	2E-02	5E-06	4.4	R Postcentral Gyrus BA40
		60	-16	24	1E-02	3E-04	3.4	R Postcentral Gyrus BA3
8	1056	22	-54	-22	3E-02	4E-09	5.8	R Cerebellum. Culmen
NM>M (GM)								
4. PreCG-R BA4: 233 foci, 15 experiments, 197 subjects (x=64, y=-14, z=8)								
1	4000	64	-16	38	7E-02	4E-30	11.4	R Postcentral Gyrus BA3
2	1152	34	20	-2	2E-02	2E-06	4.7	R Claustrum
		30	24	-12	1E-02	3E-05	4.0	R Insula BA47
M>NM (WM)								
5. IC/THA-R: 598 foci, 22 experiments, 286 subjects (x=22, y=-14, z=6), nearest grey matter: Right Thalamus.								
1	8128	22	-14	8	9E-02	2E-40	13.3	R Thalamus
		38	6	4	2E-02	4E-05	3.9	R Claustrum
		20	6	6	2E-02	4E-05	3.9	R Lentiform Nucleus
2	6848	4	-2	66	3E-02	8E-09	5.6	R Medial Frontal Gyrus BA6
		2	8	50	3E-02	3E-07	5.0	L Medial Frontal Gyrus BA6
		-8	-6	62	3E-02	5E-07	4.9	L Medial Frontal Gyrus BA6
		2	12	44	2E-02	2E-05	4.1	L Medial Frontal Gyrus BA32
		-2	2	38	2E-02	3E-05	4.1	L Cingulate Gyrus BA24
		-2	12	40	2E-02	3E-05	4.0	L Cingulate Gyrus BA32
		4	20	32	2E-02	7E-05	3.8	R Cingulate Gyrus BA32
3	5056	-24	-10	0	3E-02	1E-07	5.2	L Lentiform Nucleus
		-20	-10	2	3E-02	2E-07	5.1	L Lentiform Nucleus
		-12	0	6	2E-02	3E-06	4.6	L Thalamus
		-12	-22	4	2E-02	7E-06	4.4	L Thalamus
		-24	0	-6	2E-02	6E-05	3.9	L Lentiform Nucleus
4	1408	-28	-60	-18	3E-02	2E-07	5.0	L Cerebellum
		-32	-60	-16	3E-02	3E-07	5.0	L Cerebellum
5	1208	36	22	2	3E-02	1E-07	5.2	R Insula BA13
6	1144	-8	-60	-16	3E-02	2E-08	5.5	L Cerebellum
NM>M (WM): NA								
-	-	-	-	-	-	-	-	-
**b. FUNCTIONAL ALE ROIs**								
M>NM								
1. IFG-L BA9: 1793 foci, 83 experiments, 1238 subjects (x=-50, y=8, z=18)								
1	42352	-50	8	18	3E-01	0E+00	27.3	L Inferior Frontal Gyrus BA9
		-34	22	0	8E-02	7E-18	8.5	L Insula BA13
		-46	0	46	6E-02	4E-13	7.2	L Precentral Gyrus BA6
		-52	8	36	6E-02	8E-13	7.1	L Precentral Gyrus BA6
		-46	26	16	5E-02	2E-10	6.3	L Middle Frontal Gyrus BA46
		-46	18	-6	5E-02	6E-10	6.1	L Inferior Frontal Gyrus BA47
		-52	30	-6	4E-02	2E-08	5.5	L Inferior Frontal Gyrus BA45
		-42	38	10	4E-02	1E-07	5.2	L Middle Frontal Gyrus BA46
		-18	8	2	4E-02	7E-07	4.8	L Lentiform Nucleus
		-32	-4	54	4E-02	2E-06	4.6	L Precentral Gyrus BA6
		-36	-6	56	3E-02	3E-06	4.5	L Precentral Gyrus BA6
		-18	10	8	3E-02	3E-06	4.5	L Lentiform Nucleus
		-24	8	54	3E-02	2E-05	4.1	L Sub-Gyral BA6
2	14776	50	10	24	8E-02	4E-20	9.1	R Inferior Frontal Gyrus BA9
		34	24	-2	6E-02	8E-14	7.4	R Insula BA13
		44	2	44	3E-02	2E-05	4.1	R Middle Frontal Gyrus BA6
		44	32	20	3E-02	3E-05	4.0	R Middle Frontal Gyrus BA46
		44	30	24	3E-02	4E-05	3.9	R Middle Frontal Gyrus BA9
		52	28	24	3E-02	7E-05	3.8	R Middle Frontal Gyrus BA46
		50	18	-6	3E-02	7E-05	3.8	R Inferior Frontal Gyrus
		50	20	10	2E-02	6E-04	3.3	R Inferior Frontal Gyrus BA44
3	12568	-30	-52	42	7E-02	7E-15	7.7	No Gray Matter found
		-48	-36	42	6E-02	9E-14	7.4	L Inferior Parietal Lobule BA40
		-24	-64	50	5E-02	1E-09	6.0	L Superior Parietal Lobule BA7
		-42	-44	48	4E-02	1E-08	5.6	L Inferior Parietal Lobule BA40
4	12432	-4	16	44	6E-02	2E-12	7.0	L Medial Frontal Gyrus BA32
		6	28	34	5E-02	6E-10	6.1	R Cingulate Gyrus BA32
		-2	2	56	4E-02	3E-08	5.4	L Medial Frontal Gyrus BA6

		10	16	58	3E-02	1E-04	3.6	R Superior Frontal Gyrus BA6
5	3424	-46	-58	-10	5E-02	2E-11	6.6	L Fusiform Gyrus BA37
6	3016	46	-38	46	5E-02	1E-09	5.9	R Inferior Parietal Lobule BA40
		38	-56	48	3E-02	4E-05	4.0	R Inferior Parietal Lobule BA7
		30	-60	48	3E-02	4E-05	3.9	R Superior Parietal Lobule BA7
7	2576	18	10	4	4E-02	4E-08	5.4	R Caudate
		16	6	12	3E-02	1E-05	4.3	R Caudate
2. STG-R BA22: 1793 foci, 83 experiments, 1238 subjects (x=54, y=-10, z=4)								
1	42352	-50	8	18	3E-01	0E+00	27.3	L Inferior Frontal Gyrus BA9
		-34	22	0	8E-02	7E-18	8.5	L Insula BA13
		-46	0	46	6E-02	4E-13	7.2	L Precentral Gyrus BA6
		-52	8	36	6E-02	8E-13	7.1	L Precentral Gyrus BA6
		-46	26	16	5E-02	2E-10	6.3	L Middle Frontal Gyrus BA46
		-46	18	-6	5E-02	6E-10	6.1	L Inferior Frontal Gyrus BA47
		-52	30	-6	4E-02	2E-08	5.5	L Inferior Frontal Gyrus BA45
		-42	38	10	4E-02	1E-07	5.2	L Middle Frontal Gyrus BA46
		-18	8	2	4E-02	7E-07	4.8	L Lentiform Nucleus
		-32	-4	54	4E-02	2E-06	4.6	L Precentral Gyrus BA6
		-36	-6	56	3E-02	3E-06	4.5	L Precentral Gyrus BA6
		-18	10	8	3E-02	3E-06	4.5	L Lentiform Nucleus
		-24	8	54	3E-02	2E-05	4.1	L Sub-Gyral BA6
2	14776	50	10	24	8E-02	4E-20	9.1	R Inferior Frontal Gyrus BA9
		34	24	-2	6E-02	8E-14	7.4	R Insula BA13
		44	2	44	3E-02	2E-05	4.1	R Middle Frontal Gyrus BA6
		44	32	20	3E-02	3E-05	4.0	R Middle Frontal Gyrus BA46
		44	30	24	3E-02	4E-05	3.9	R Middle Frontal Gyrus BA9
		52	28	24	3E-02	7E-05	3.8	R Middle Frontal Gyrus BA46
		50	18	-6	3E-02	7E-05	3.8	R Inferior Frontal Gyrus
		50	20	10	2E-02	6E-04	3.3	R Inferior Frontal Gyrus BA44
3	12568	-30	-52	42	7E-02	7E-15	7.7	No Gray Matter found
		-48	-36	42	6E-02	9E-14	7.4	L Inferior Parietal Lobule BA40
		-24	-64	50	5E-02	1E-09	6.0	L Superior Parietal Lobule BA7
		-42	-44	48	4E-02	1E-08	5.6	L Inferior Parietal Lobule BA40
4	12432	-4	16	44	6E-02	2E-12	7.0	L Medial Frontal Gyrus BA32
		6	28	34	5E-02	6E-10	6.1	R Cingulate Gyrus BA32
		-2	2	56	4E-02	3E-08	5.4	L Medial Frontal Gyrus BA6
		10	16	58	3E-02	1E-04	3.6	R Superior Frontal Gyrus BA6
5	3424	-46	-58	-10	5E-02	2E-11	6.6	L Fusiform Gyrus BA37
6	3016	46	-38	46	5E-02	1E-09	5.9	R Inferior Parietal Lobule BA40
		38	-56	48	3E-02	4E-05	4.0	R Inferior Parietal Lobule BA7
		30	-60	48	3E-02	4E-05	3.9	R Superior Parietal Lobule BA7
7	2576	18	10	4	4E-02	4E-08	5.4	R Caudate
		16	6	12	3E-02	1E-05	4.3	R Caudate
3. STG-L BA22: 588 foci, 36 experiments, 555 subjects (x=-58, y=-46, z=16)								
1	8320	-58	-44	16	1E-01	0E+00	16.8	L Superior Temporal Gyrus BA22
2	4272	-44	24	20	3E-02	6E-07	4.9	L Middle Frontal Gyrus BA46
		-44	14	22	2E-02	2E-06	4.6	L Inferior Frontal Gyrus BA9
		-42	12	28	2E-02	6E-06	4.4	L Inferior Frontal Gyrus BA9
		-40	2	44	2E-02	2E-05	4.1	L Precentral Gyrus BA6
		-44	6	20	2E-02	3E-05	4.1	L Inferior Frontal Gyrus BA9
		-40	0	32	2E-02	3E-05	4.0	L Precentral Gyrus BA6
3	2368	58	-38	4	3E-02	2E-07	5.0	R Middle Temporal Gyrus BA22
		50	-38	6	2E-02	1E-05	4.2	R Superior Temporal Gyrus BA41
		56	-50	6	2E-02	1E-05	4.2	R Middle Temporal Gyrus BA21
		62	-42	16	2E-02	1E-04	3.7	R Superior Temporal Gyrus BA13
		48	-32	10	2E-02	3E-04	3.4	R Superior Temporal Gyrus BA41
4	2272	34	20	0	3E-02	7E-07	4.8	R Claustrum
		48	16	4	2E-02	3E-05	4.0	R Precentral Gyrus BA44
		52	26	-2	2E-02	2E-04	3.5	R Inferior Frontal Gyrus BA45
5	2272	-42	16	2	3E-02	2E-09	5.9	L Insula BA13
		-52	24	6	2E-02	1E-05	4.2	L Inferior Frontal Gyrus BA45
6	1680	-6	12	50	3E-02	2E-07	5.1	L Medial Frontal Gyrus BA6
NM>M								
4. IPL-L BA40: 565 foci, 28 experiments, 321 subjects (x=-50, y=-36, z=56)								
1	10416	-48	-36	54	1E-01	0E+00	14.6	L Inferior Parietal Lobule BA40

		-40	-12	56	2E-02	1E-07	5.2	L Precentral Gyrus BA4
		-34	-28	64	2E-02	8E-05	3.8	L Postcentral Gyrus BA3
2	4808	-2	6	54	3E-02	2E-09	5.9	L Medial Frontal Gyrus BA6
		-4	-6	68	1E-02	7E-04	3.2	L Medial Frontal Gyrus BA6
3	1720	52	20	-4	2E-02	1E-07	5.2	R Inferior Frontal Gyrus BA47
4	1376	-58	4	30	2E-02	3E-06	4.5	L Precentral Gyrus BA6
		-50	10	30	2E-02	5E-05	3.9	L Inferior Frontal Gyrus BA9
5. PreCG-L BA6: 2068 foci, 108 experiments, 1982 subjects (x=-48, y=8, z=36)								
1	33856	-48	8	36	4E-01	0E+00	29.9	L Precentral Gyrus BA6
		-32	22	0	1E-01	6E-28	10.9	L Claustrum
		-48	24	26	6E-02	2E-12	6.9	L Middle Frontal Gyrus BA9
		-52	10	16	5E-02	4E-09	5.8	L Inferior Frontal Gyrus BA44
		-50	12	2	5E-02	6E-09	5.7	L Insula BA13
		-26	-4	58	5E-02	4E-08	5.4	L Middle Frontal Gyrus BA6
		-50	28	4	4E-02	2E-07	5.1	L Inferior Frontal Gyrus BA45
		-46	24	-6	4E-02	9E-07	4.8	L Inferior Frontal Gyrus BA47
		-50	14	-14	4E-02	8E-06	4.3	L Superior Temporal Gyrus BA38
2	15696	-24	-66	46	7E-02	6E-14	7.4	L Precuneus BA7
		-36	-52	46	7E-02	1E-13	7.3	L Inferior Parietal Lobule BA40
		-44	-36	48	4E-02	6E-07	4.9	L Inferior Parietal Lobule BA40
3	14160	-4	14	50	1E-01	9E-27	10.6	L Superior Frontal Gyrus BA6
		6	28	36	6E-02	2E-10	6.3	R Cingulate Gyrus BA32
		-6	24	32	4E-02	4E-06	4.5	L Cingulate Gyrus BA32
		-4	32	28	3E-02	2E-05	4.2	L Cingulate Gyrus BA32
4	11032	50	10	26	8E-02	1E-16	8.2	R Inferior Frontal Gyrus BA9
		48	36	24	4E-02	2E-07	5.1	R Middle Frontal Gyrus BA9
5	5952	40	-50	48	5E-02	9E-10	6.0	R Inferior Parietal Lobule BA40
		34	-60	46	5E-02	2E-09	5.9	R Precuneus BA19
		46	-38	50	4E-02	6E-06	4.4	R Inferior Parietal Lobule BA40
		26	-68	48	3E-02	2E-05	4.1	R Precuneus BA7
		24	-70	52	3E-02	2E-05	4.1	R Precuneus BA7
		26	-58	60	3E-02	6E-04	3.2	R Superior Parietal Lobule BA7
6	5656	34	22	-2	9E-02	6E-20	9.1	R Insula
		52	18	-6	3E-02	3E-05	4.0	R Inferior Frontal Gyrus
7	4464	-44	-60	-16	5E-02	1E-09	6.0	L Fusiform Gyrus BA37
		-40	-72	-12	5E-02	1E-08	5.6	L Fusiform Gyrus BA19
		-50	-54	0	3E-02	2E-04	3.5	L Middle Temporal Gyrus BA37
8	3864	-8	-12	8	5E-02	3E-09	5.8	L Thalamus
		-18	8	4	3E-02	4E-05	3.9	L Lentiform Nucleus
9	1800	32	0	50	4E-02	4E-07	5.0	R Middle Frontal Gyrus BA6

ALE, anatomic likelihood estimation; M, musicians; NM, non-musicians; GM, gray matter; WM, white matter; BA, Brodmann area; ROIs, regions-of-interest; P, p-value; Z, peak z-value; R, right; L, left. **ROIs**: IFG, inferior frontal gyrus; IPL, inferior parietal lobule; IC, internal capsule; PostCG, postcentral gyrus (primary somatosensory cortex or S1); PreCG, precentral gyrus (primary motor cortex or M1); STG, superior temporal gyrus (primary auditory cortex). Music-related ROIs were created in Mango (http://rii.uthscsa.edu/mango//userguide.html) with a 5mm-radius sphere. Last search in Sleuth, 10.10.2021 (http://www.brainmap.org/sleuth/); NA, not enough available observations.

Supplementary Table 5. Functional characterization of brain regions resulted from structural and functional ALE meta-analyses.

**a. STRUCTURAL ALE ROIs**	
M>NM (GM)	
1. STG-R BA13: 992 foci, 46 experiments, 593 subjects (x=50, y=-20, z=8)	
Action	Execution, speech, motor learning, preparation
Cognition	Attention, language, semantics speech, syntax, explicit memory, working memory, music, reasoning
Emotion	Anger, anxiety, sadness, positive emotion, happiness, reward/gain
Interoception	Sexuality
Perception	Audition, somesthesis, pain, vision
Paradigms	Affective words, classical conditioning, counting/calculation, delayed match to sample, emotion induction, face discrimination, finger tapping/button pressing, flexion/extension, go/no-go, meditation, music comprehension, music production, n-back, naming overt, pain discrimination, passive listening, passive viewing, phonological discrimination, pitch discrimination, reading overt, reasoning/problem solving, recitation/repetition, reward, semantic discrimination, sequence recall/learning, sexual arousal, syntactic discrimination, tone discrimination, word generation
2. STG-L BA41: 1428 foci, 71 experiments, 961 subjects (x=-56, y=-20, z=2)	
Action	Execution, speech, imagination, inhibition, motor learning, observation, preparation
Cognition	Attention, language, orthography, phonology, semantics, speech, syntax, explicit memory, working memory, music, reasoning, spatial
Emotion	Disgust, fear, guilt, sadness, happiness, valence
Interoception	Sexuality, thermoregulation
Perception	Audition, pain, vision, motion, shape
Paradigms	Cued explicit recognition, emotion induction, face discrimination, film viewing, finger tapping/button pressing, free list word recall, go/no-go, imagined objects/scenes, mental rotation, music comprehension, music production, naming, oddball discrimination, orthographic discrimination, pain discrimination, paired associate recall, passive listening, passive viewing, phonological discrimination, pitch discrimination, reading, reasoning/problem solving, recitation/repetition, semantic discrimination, sequence recall/learning, sexual arousal, tone discrimination, visual motion, visuospatial attention, word generation
3. PostCG-R BA2: 437 foci, 22 experiments, 310 subjects (x=54, y=-22, z=44)	
Action	Execution, motor learning
Cognition	Attention, somatic
Emotion	-
Interoception	Respiration regulation
Perception	Audition, somesthesis, pain, vision, colour, shape
Paradigms	Chewing/swallowing, drawing, face discrimination, finger tapping/button pressing, flanker, flexion/extension, go/no-go, hypercapnia, motor learning, oddball discrimination, pain discrimination, passive listening, passive viewing, pursuit/tracking, saccades, tactile discrimination, tone discrimination, transcranial magnetic stimulation, visuospatial attention, writing
NM>M (GM)	
4. PreCG-R BA4: 233 foci, 15 experiments, 197 subjects (x=64, y=-14, z=8)	
Action	Execution, inhibition, observation, preparation
Cognition	Attention, semantics, speech, social cognition, temporal
Emotion	Sadness, happiness
Interoception	-
Perception	Gustation, somesthesis, pain, vision
Paradigms	Competition/cooperation, deception, face discrimination, film viewing, finger tapping/button pressing, flanker, flexion/extension, go/no-go, grasping, imagined movement, isometric force, pain discrimination, passive viewing, recitation/repetition, taste, transcranial magnetic stimulation, video games, visuospatial attention
M>NM (WM)	
5. IC-R: 598 foci, 22 experiments, 286 subjects (x=22, y=-14, 6), nearest grey matter: Right Thalamus.	
Action	Execution, speech, imagination
Cognition	Attention, orthography, explicit memory, working memory, reasoning
Emotion	Negative emotion, happiness, reward
Interoception	Thermoregulation
Perception	Audition, olfaction, pain
Paradigms	Counting/calculation, cued explicit recognition/recall, emotion induction, episodic recall, face discrimination, finger tapping/button pressing, flexion/extension, free list word recall, go/no-go, imagined movement, isometric force, n-back, olfactory discrimination, pain discrimination, passive listening, reading, recitation/repetition, reward, tone discrimination
NM>M (WM): NA	
-	

**b. FUNCTIONAL ALE ROIs**	
M>NM	
1. IFG-L BA9: 1793 foci, 83 experiments, 1238 subjects (x=-50, y=8, z=18)	
Action	Execution, speech, imagination, inhibition, observation, preparation
Cognition	Attention, language, orthography, phonology, semantics, speech, syntax, explicit memory, working memory, music, reasoning, social cognition, spatial
Emotion	Negative emotion, anger, fear, reward
Interoception	-
Perception	Audition, gustation, somesthesis, pain, vision, motion, shape
Paradigms	Chewing/swallowing, counting/calculation, cued explicit recognition/recall, delayed match to sample, driving, encoding, face discrimination, figurative language, film viewing, finger tapping/button pressing, flexion/extension, gambling, go/no-go, imagined movement, imagined objects/scenes, lexical decision, magnitude comparison, meditation, mental rotation, music comprehension, music production, n-back, naming, orthographic discrimination, pain discrimination, paired associate recall, passive listening, passive viewing, phonological discrimination, reading, reasoning/problem solving, recitation/repetition, reward, saccades, semantic discrimination, sequence recall/learning, tactile discrimination, theory-of-mind, tone discrimination, visual object identification, Wisconsin Card Sorting Test, word generation, word stem completion
2. STG-R BA22: 1793 foci, 83 experiments, 1238 subjects (x=54, y=-10, z=4)	
Action	Speech, imagination, inhibition, motor learning
Cognition	Attention, phonology, speech, music, reasoning, spatial
Emotion	Reward
Interoception	Hunger, osmoregulation, thermoregulation, thirst
Perception	Audition, gustation, somesthesis, pain, vision
Paradigms	Acupuncture, counting/calculation, emotion induction, encoding, face discrimination, film viewing, finger tapping/button pressing, flexion/extension, gambling, go/no-go, hunger, imagined movement, imagined objects/scenes, multi-tasking, music comprehension, music production, pain discrimination, passive listening, passive viewing, phonological discrimination, pitch discrimination, reading, reasoning/problem solving, reward, semantic discrimination, sequence recall/learning, taste, thirst induction, tone discrimination
3. STG-L BA22: 588 foci, 36 experiments, 555 subjects (x=-58, y=-46, z=16)	
Action	Execution, speech, imagination, inhibition, observation
Cognition	Attention, language, phonology, semantics, speech, explicit memory, music, reasoning, social cognition
Emotion	Disgust, embarrassment, positive emotion
Interoception	Sexuality
Perception	Audition, somesthesis, pain, vision
Paradigms	Acupuncture, affective pictures, controlled breathing, cued explicit recognition/recall, divided auditory attention, emotion induction, emotional body language perception, encoding, face discrimination, finger tapping/button pressing, go/no-go, music comprehension, music production, oddball discrimination, pain discrimination, paired associate recall, passive viewing, phonological discrimination, pitch discrimination, reading, reasoning/problem solving, recitation/repetition, semantic discrimination, Stroop-color, theory-of-mind, tone discrimination, visual motion, visuospatial attention, word generation
NM>M	
4. IPL-L BA40: 565 foci, 28 experiments, 321 subjects (x=-50, y=-36, z=56)	
Action	Execution, speech, imagination, inhibition, motor learning, preparation
Cognition	Attention, orthography, phonology, semantics, explicit memory, working memory, music, reasoning, social cognition, spatial
Emotion	Negative emotion, anxiety, reward
Interoception	-
Perception	Audition, somesthesis, pain, vision, shape
Paradigms	Classical conditioning, counting/calculation, cued explicit recognition/recall, emotion induction, film viewing, finger tapping/button pressing, fixation, flexion/extension, go/no-go, imagined movement, imagined objects/scenes, mental rotation, motor learning, multi-tasking, object discrimination, pain discrimination, phonological discrimination, reading, reasoning/problem solving, recitation/repetition, reward, semantic discrimination, Stroop colour, tactile discrimination, task switching, tone discrimination, visual object identification, visuospatial attention
5. PreCG-L BA6: 2068 foci, 108 experiments, 1982 subjects (x=-48, y=8, z=36)	
Action	Execution, speech, imagination, inhibition, motor learning, observation, preparation
Cognition	Attention, language, orthography, phonology, semantics, speech, syntax, explicit memory, working memory, music, reasoning, social cognition, somatic, spatial, temporal
Emotion	Negative emotion, anger, disgust, far, positive emotion, happiness, reward
Interoception	Hunger, sexuality, sleep
Perception	Audition, gustation, somesthesis, vision, color, motion, shape
Paradigms	Affective pictures, anti-saccades, chewing/swallowing, counting/calculation, cued explicit recognition/recall, deception, delayed match to sample, divided auditory attention, drawing, driving, emotion induction, encoding, episodic recall, estimation, face discrimination, figurative language, film viewing, finger tapping/button pressing, flanker, gambling, go/no-go, hunger, imagined movement, imagined objects/scenes, mental rotation, multi-tasking, music comprehension,

	n-back, naming, oddball discrimination, orthographic discrimination, paired associate recall, phonological discrimination, pitch discrimination, pursuit, reading, reasoning/problem solving, recitation, reward, saccades, semantic discrimination, Stroop, tactile discrimination, task switching, taste, theory of mind, tone discrimination, visual object identification, visual pursuit, visuospatial attention, Wisconsin card sorting, word generation

ALE, anatomic likelihood estimation; M, musicians; NM, non-musicians; GM, grey matter; WM, white matter; BA, Brodmann area; ROIs, regions-of-interest; P, p-value; Z, peak z-value; R, right; L, left. **ROIs**: IFG, inferior frontal gyrus; IPL, inferior parietal lobule; IC, internal capsule; PostCG, postcentral gyrus (primary somatosensory cortex or S1); PreCG, precentral gyrus (primary motor cortex or M1); STG, superior temporal gyrus (primary auditory cortex). Music-related ROIs were created in Mango (http://rii.uthscsa.edu/mango//userguide.html) with a 5mm-radius sphere. Last search in Sleuth, 10.10.2021 (http://www.brainmap.org/sleuth/); NA, not enough available observations.

Supplementary Table 6. FSN robustness assessment of brain regions resulted from structural and functional ALE meta-analyses.

Cluster number	Volume (mm³)	MNI coordinates x	y	z	ALE	Label (Side, region)	Contributing studies (k)	FSN
**a. STRUCTURAL ALE ROIs**								
M>NM (GM): 133 foci, 20 experiments, 1071 subjects, minimum FSN = 6								
1	912	50	-20	8	2E-02	R Superior Temporal Gyrus BA13	5	10
2	784	-56	-20	2	2E-02	L Superior Temporal Gyrus BA41	3	10
3	536	54	-22	44	2E-02	R Postcentral Gyrus BA2 (S1)	3	<6
NM>M (GM): 22 foci, 8 experiments, 305 subjects, minimum FSN = 3								
4	520	64	-14	38	1E-02	R Precentral Gyrus BA4 (MI)	2	<3
M>NM (WM): 22 foci, 5 experiments, 139 subjects, minimum FSN = 2								
5	792	22	-14	6	1E-02	R Thalamus, Internal Capsule	3	18
NM>M (WM): NA								
-	-	-	-	-	-	-	-	-
**b. FUNCTIONAL ALE ROIs**								
M>NM: 354 foci, 34 experiments, 979 subjects, minimum FSN = 10								
1	3080	-50	8	18	3E-02	L Inferior Frontal Gyrus BA9	24	90
2	1136	54	-10	4	2E-02	R Superior Temporal Gyrus BA22	7	70
3	920	-58	-46	16	2E-02	L Superior Temporal Gyrus BA22	7	<10
NM>M: 144 foci, 12 experiments, 286 subjects, minimum FSN = 4								
4	912	-50	-36	56	1E-02	L Inferior Parietal Lobule BA40	5	8
5	736	-48	8	36	2E-02	L Precentral Gyrus BA6	5	<4

FSN, Fail-Safe N analysis; NA, not enough available observations.

[Citations of included studies]

Abbreviations

ACC	anterior cingulate cortex
AF	arcuate fasciculus
AnG	angular gyrus
CalC	calcarine cortex
CC	corpus callosum
CAU	caudate
CLAU	claustrum
CRBL	cerebellum
CST	corticospinal tract
CUN	cuneus
DLPFC	dorsolateral prefrontal cortex
EC	entorhinal cortex
Fmaj	forceps major
Fmin	forceps minor
FO	frontal operculum
FusG	fusiform gyrus
GP	globus pallidus
HG	Helsch's gyrus
HIPP	hippocampus
IC	internal capsule
IF	inferior colliculus
IFG	inferior frontal gyrus
IFOF	inferior fronto-occipital fasciculus
ILF	inferior longitudinal fasciculus
INS	insula
IPL	inferior parieal lobule
ITG	inferior temporal gyrus
LG	lingual gyrus
LOC	lateral occipital cortex
MB	midbrain
MCC	middle cingulate cortex
MCP	middle cerebellar peduncle
MedFG	medial frontal gyrus
MidFG	middle frontal gyrus
MidTG	middle temporal gyrus
OFC	orbitofrontal cortex
PaHIPP	parahippocampal gyrus
PCC	posterior cingulate cortex
PCN	precuneus
PMC	premotor cortex
PO	parietal operculum
PostCG	postcentral gyrus (primary somatosensory cortex or SI)
PP	planum polare
PreCG	precentral gyrus (primiary motor cortex or M1)
PT	planum temporale
PUT	putamen
RN	red nucleus
SCP	superior cerebellar peduncle
SFG	superior frontal gyrus
SII	secondary somatosensory cortex
SLF	superior longitudial fasciculus
SMA	supplementary motor area
SMG	supramarginal gyrus
SPL	superior parietal lobule
STG	superior temporal gyrus
STS	superior temporal sulcus
THA	thalamus
TP	temporal pole
TPG	temporoparietal junction
VER	Vermis

References

1. Poldrack, R. A. *et al.* Guidelines for reporting an fMRI study. *Neuroimage* **40**, 409–414 (2008).
2. Nichols, T. E. *et al.* Best practices in data analysis and sharing in neuroimaging using MRI. *Nat. Neurosci.* **20**, 299–303 (2017).
3. Laird, A. R. *et al. User Manual for Sleuth 2.0*.
4. Eickhoff, S. B., Bzdok, D., Laird, A. R., Kurth, F. & Fox, P. T. Activation likelihood estimation meta-analysis revisited. *Neuroimage* **59**, 2349–2361 (2012).
5. Laird, A. R. *et al.* Networks of task co-activations. *Neuroimage* **80**, 505–514 (2013).
